# Nanocomposite Hydrogels: Advances in Nanofillers Used for Nanomedicine

**DOI:** 10.3390/gels4030075

**Published:** 2018-09-06

**Authors:** Arti Vashist, Ajeet Kaushik, Anujit Ghosal, Jyoti Bala, Roozbeh Nikkhah-Moshaie, Waseem A. Wani, Pandiaraj Manickam, Madhavan Nair

**Affiliations:** 1 Department of Immunology & Nano-Medicine, Institute of NeuroImmune Pharmacology, Centre for Personalized Nanomedicine, Herbert Wertheim College of Medicine, Florida International University, Miami, FL 33199, USA; akaushik@fiu.edu (A.K.); jbala@fiu.edu (J.B.); rnikkhah@fiu.edu (R.N.-M.); 2 School of Biotechnology, Jawaharlal Nehru University, New Delhi 110067, India; anuj.ghosal@gmail.com; 3 Department of Chemistry, Govt. Degree College Tral, Kashmir, J&K 192123, India; waseemorg@gmail.com; 4 Electrodics and Electrocatalysis Division, CSIR-Central Electrochemical Research Institute, Karaikudi 630006, Tamil Nadu, India; mpraj88@gmail.com

**Keywords:** biomaterials, nanocomposite hydrogels, nanomedicine, biomedical applications, carbon nanotubes, pH responsive, biosensors

## Abstract

The ongoing progress in the development of hydrogel technology has led to the emergence of materials with unique features and applications in medicine. The innovations behind the invention of nanocomposite hydrogels include new approaches towards synthesizing and modifying the hydrogels using diverse nanofillers synergistically with conventional polymeric hydrogel matrices. The present review focuses on the unique features of various important nanofillers used to develop nanocomposite hydrogels and the ongoing development of newly hydrogel systems designed using these nanofillers. This article gives an insight in the advancement of nanocomposite hydrogels for nanomedicine.

## 1. Introduction

The most emerging field of nanomedicine has come up with diverse biomedical applications ranging from optical devices, biosensors, advanced drug delivery systems, and imaging probes. In this regard, hydrogels have been intensively studied and explored [1,2]. Nanomedicine is a developing field, which deals with medical applications related to nanomaterials and biological devices. Nanomedicine showed great advancements and utilizes the change in the functionalities of nanomaterials with synergism to biological molecules. This combination of nanomaterials and biological molecules has led to the invention of unique devices, analytical tools, many novel physical therapies, and vehicles for proteins and enzymes. It is worth noting that the pace of investigation going on around the globe for hydrogels in nanomedicine is very high. The importance of nanomedicine in market has been demanding due to the unique characteristic properties arising from the application of nanotechnology. A fundamental understanding of the term “nano”, precisely with reference to nanofillers and nanoparticles, corresponds to particles with sizes ranging between 1 to 100 nm with a surrounding interfacial layer. This layer is an important and integral part of nanorange materials. This size specification affects all the properties and features of the materials.

To begin with, hydrogels are the crosslinked 3D-networks of hydrophilic polymers, having great tendency to absorb large amounts of water inside them. The unique features of hydrogels include their soft porous structures, high water content, biocompatibility, and ability to absorb physiological fluids. Moreover, the presence of specific functional groups on the backbone chains of the polymeric matrices are key factors to decide their response in various environments. This unique ability of the hydrogels to respond to external stimuli makes them intelligent carriers [3]. Recent research shows that the development of nanomaterials, either in the form of inorganic nanoframeworks, nanogels [4,5] or nanocomposite hydrogels [6]; is the future of up-and-coming nanomedicine technology.

Recent trends have suggested the development of hydrogels with the functionality of inorganic components. There is a great difference between a nanocarrier alone and when it acts as a drug delivery system. The capacity for drug encapsulation, its stability, and effectiveness of delivery vehicles are excessively enhanced when we consider nanocomposite hydrogels as drug delivery systems.

**Figure 1:**
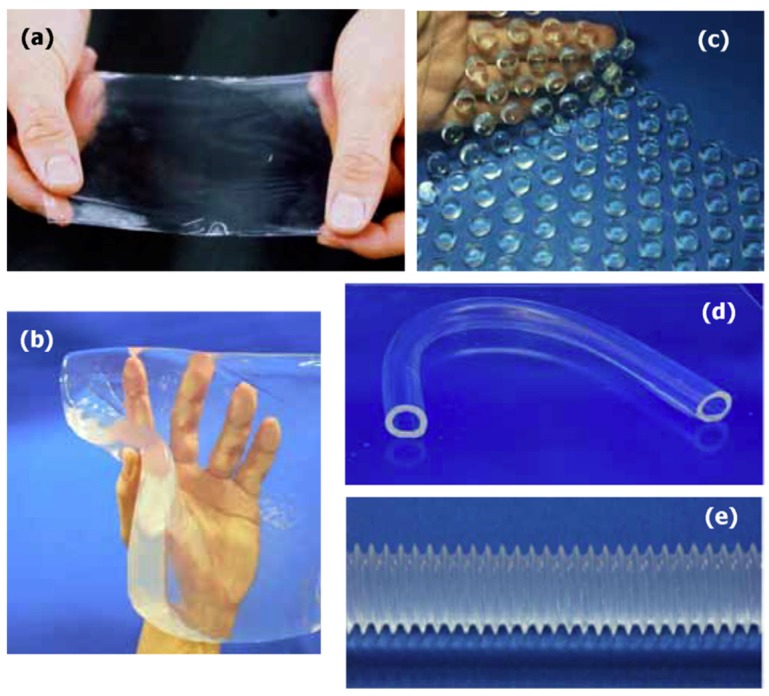
Nanocomposite hydrogels gels with various shapes: (**a**) thin film, (**b**) sheet, (**c**) uneven sheet, (**d**) hollow tube, and (**e**) bellows. Reprinted with permission from Reference [8]. Copyright 2007 Elsevier.

The development of inorganic-organic frameworks with soft hydrogel networks is the demand of today’s materials. Clay nanocomposite hydrogels were innovative gels developed by Haraguchi in 2002 [7]. These nanocomposite hydrogels exhibited superior properties, including mechanical and swelling/deswelling features when compared to conventional hydrogels. This study came to be a breakthrough in the world of nanocomposite hydrogels. Nanocomposite hydrogels are simply the cross-linked three-dimensional networks formed in the presence of nanostructures. These structures are formed either by physical or chemical crosslinking. Physical linkages are formed through hydrogen bonding or via hydrophobic or ionic interactions. Chemical crosslinking occurs by the formation of covalent bonds, which are very strong. These nanocomposite hydrogels are capable of changing their properties depending upon external stimuli. The nanoparticles are added to the hydrogel matrix either by absorbing these nanoparticles inside the matrix or dispersing them homogeneously inside the hydrogel matrix and also by entrapping nanoparticles inside the hydrogel matrix. These nanoparticles act as nanofillers inside the matrix, which causes great enhancement in intrinsic and extrinsic properties of the hydrogel matrix. The nanofiller addition to the hydrogel matrix contributes to the unique properties of hydrogels and enhances their applications in microfluidics, sensors, and actuators. Moreover, the nanocomposite hydrogels are being applied in catalysis, development of membranes used for separation, water purification, and other various biomedical applications. This diversity exists because these nanocomposites can be designed in various forms, such as thick and thin membranes, uneven sheets, hollow tubes, and bellows (Figure 1).

## 2. Various Nanofillers Used for Designing Nanocomposite Hydrogels

**Figure 2:**
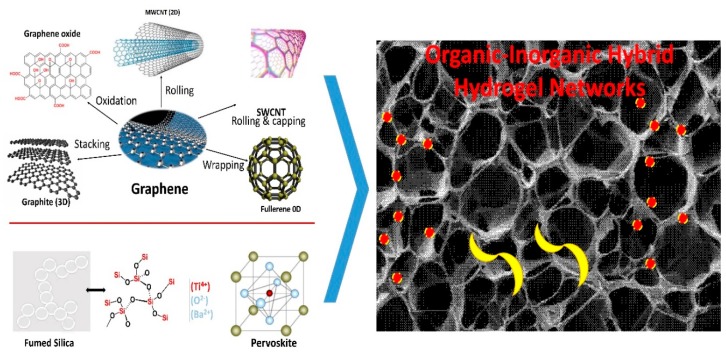
Illustration of potential nanofillers such as 0D (fullerene, C60), 1D small-walled carbon nanotubes & Multi-walled carbon nanotubes (SWCNTs & MWCNTs), 2D (graphene, graphene oxide or functionalized graphene), and 3D (graphite, silicate, BaTiO_3_), which are used to synthesize nanocomposite hydrogel networks based on organic-inorganic-organic hybrid nanocomposite chemistry.

Hydrogels are covalently or physically crosslinked networks and hence, usually have poor mechanical strength, elastic modulus, tensile strength, etc. To overcome these drawbacks, which restrict the high end applications of hydrogels, nanofillers can be introduced into these networks as discussed in next section. Many known organic and inorganic nanostructures are being incorporated as nanofillers in hydrogel matrices to obtain nanocomposite hydrogels. The synergism between the crosslinked polymeric network and the various nanofillers (Figure 2) give rise to astonishing enhancement of the characteristic properties of the nanocomposite hydrogel systems. The physical and covalent interactions come up with novel and value-added properties. Various properties are affected, including swelling, by the addition of nanofillers. The swelling capacity can be limited by modulating the concentration of inorganic nanofillers for suitable swelling features. It is noteworthy that while considering nanocomposite hydrogels, when any nanofiller is added to the hydrogel matrix, the swelling of the system gets affected to a great extent and thus, they exhibit far more superior properties and applications than the conventional systems. The modulation of the content and methodological approaches for the synthesis of nanocomposite hydrogels has created advanced materials for personalized nanomedicine. In the next sections, we will discuss a few important nanofillers used to develop nanocomposite hydrogels.

### 2.1. Graphene Based Nanocomposite Hydrogels

Graphene has a unique two-dimensional structure with excellent mechanical, physico-chemical, thermal, optical, electrical, and biomedical properties [9,10]. Graphene can be easily converted into graphene oxide (GO) with functionalities like carboxylic acid, epoxide, and hydroxyl groups in the plane and reduced graphene oxide (rGO). These derivatives of the graphene family possess a combination of hydrophilic as well as hydrophobic (π–π interactions) which provides them with colloidal stability, pH-dependent surface charges, dipole interactions, hydrogen bonding, and other surface reactions along with non-covalent functionalization. With their amphiphilic nature and the ability to be functionalized, graphene, rGO, and GO-functionalized conjugates have shown high drug entrapment ability with particular sensitivity for hydrophobic drug molecules [9]. Thus, these nanofillers are extensively employed when designing nanocomposite hydrogels for imbibing hydrophobic drugs [11]. Hydrogels have been recently employed by using hierarchical graphene oxide sheets and peptide assemblies for on-demand drug release applications [12]. Furthermore, hybrid systems have been tested, which has demonstrated that when 2D material with diverse bio-functionalities is incorporated inside the 3D hydrogel matrix, it may be used as new cell culture system [13]. Additionally, the expression of specific biomarkers has been enhanced by using the hybrid systems. Recently, the research group of Patel et al. demonstrated the use of oxide/polypeptide thermo-gel (GO/P) and showed that when insulin is adhered to GO, it can be supplied to the stem cells in a much more sustained manner. Thus, these kinds of systems hold great potential as 3D cell culture matrices, which utilize the surface properties of the 2D materials and then the stem cells are transcribed by the 3D culture systems [14]. One of the interesting study carried out by Sahu et al. reported a thermo-sensitive injectable hydrogel system based on nano-sized GO with no severe acute cytotoxicity [15]. The main driving force for the gelation of disperse graphene sheets, pluronic block copolymers and cyclodextrin was hydrogen bonding, π–π interaction, or electrostatic interaction. Another injectable and mechanically robust 4-arm polypropylene oxide–polyethylene oxide/Graphene oxide (PPO–PEO/GO) composite hydrogel with high water dispersibility promised its use as a biomaterial platform or drug carrier [16]. Potential anti-thrombogenic and scaffold materials with hemocompatibility have been prepared by radical copolymerization. The resultant GO-based hydrogels were heparin-mimicking with thin pore walls, high porosity, and anti-platelet adhesion abilities as well anticoagulant abilities [17]. Electro-active scaffolds and DNA–graphene based nanocomposite hydrogel systems have great biomedical usability with a high self-healing function, excellent environmental stability, and adsorption capability [18,19]. Other interesting studies have involved the use of graphene as a nanofiller. Such studies have highlighted enhanced mechanical strength and tensibility when graphene is added to the hydrogel matrix [20,21]. One study showed that graphene oxide, when added in the GelMA hydrogels, enhanced the mechanical strength and caused a reduction in UV-induced cell damage. The overall characteristics needed for 3D encapsulation were restored. The study highlighted the use of multi-stacking approaches, which led to an improved and easier synthesis strategy for the construction of the complex artificial tissues [22]. Moreover, GO has shown potential applications for injectable hydrogel for gene delivery [23].

A very innovative study by Khademhosseini et al. [23] showed a non-viral gene delivery system using polyethylenimine (PEI) and functionalized by GO and vascular endothelial growth factor-165 (VEGF) pro-angiogenic gene for myocardial therapeutic applications. The group used a rat model to test the therapeutic effects of the hydrogel in an acute myocardial infarction. The study demonstrated that there was a significant increase in myocardial capillary density and reduction in the scar area. High cardiac performance in echocardiography was observed after 14 days post-infection. The injected sites showed no inflammation and serum cytokines levels. Thus, these kinds of studies support the effectiveness of an injectable hydrogel-based system when used in a gene therapy system for ischemic heart diseases.

### 2.2. Metallic Nanoparticles Based Nanocomposite Hydrogels

The synergism of metallic nanoparticles and hydrogel matrices has delivered outstanding performances for the resultant nanocomposite hydrogels [24]. Metallic nanoparticles affect the hydrogel depending on the type of interaction i.e., strong or weak. A weaker interaction has little effect on the mechanical properties of the nanocomposite hydrogels but can improve conductivity, stimuli responses, and antimicrobial properties i.e., the addition of indigenous properties of metal nanoparticles. On the other hand, a stronger interaction between these two components enhances the swelling behavior, localized surface plasmon resonance (allowing the material to be analyzed by UV-Vis, Raman spectra, etc.), sensitivity towards-pH, heat, and electricity, etc. Gold nanoparticle-based hydrogels possess thermo-switchable electronic properties, enhanced electrochemical properties, and can be used as a light-responsive hydrogel for drug delivery, sensors, catalysts, cancer therapy, and tissue engineering purposes, etc. [24,25,26,27,28]. Furthermore, the addition of silver nanoparticles enrich the electronic, antibacterial, and antifungal properties of hydrogels [29,30].

Hydrogels with metallic nanoparticles such as Fe, Co, Ni, and their oxides like Fe_3_O_4_, Fe_2_O_3_, CoFe_2_O_3_, FePt, CoPt, etc., which are magnetic in nature, have been found to act as excellent tunable extracellular matrices. Diamagnetic metallic nanoparticles of silver, gold, calcium, and their oxides have also been used for the reinforcement of hydrogel structures. However, the remote controlling ability of magnetic hydrogels have drawn research attention in the development of new and flexible magnetic hydrogels, [31] which can be appropriately positioned by a magnetic field to create complex cellular clusters [32]. They have mostly been used in tissue engineering, drug delivery, separation techniques, image enhancement, remote controlled actuators, stem cell treatment, and as scaffolds for tissue engineering. Zhang et al. formulated a hybrid hydrogel for potential cartilage tissue engineering using type II collagen, hyaluronic acid (HA), polyethylene glycol (PEG), and Fe_3_O_4_ magnetic nanoparticles with cyto-compatibility with bone marrow-derived mesenchymal stem cells [33]. These magnetic gels showed a similar microstructure and chemistry to hyaline cartilage. Spatiotemporal regenerative growth factors and mechanical stimuli are of critical importance for tissue regeneration, yet not found in most of the classical scaffolds used [34]. Magnetic DNA hydrogel with unique properties have been formulated using “DNA-modified magnetic nanoparticles” in DNA hydrogel networks. It is also known that an external magnetic field can remotely trigger changes in the shapes of hydrogels. Furthermore, various stimuli like temperature, pH, enzymes, and a magnetic field triggers a gelation reaction [35]. Near-infrared (NIR) responsive nanocomposite hydrogels using metallic nanofillers are in high demand [36]. Thus, metallic nanoparticles hold great potential as a nanofiller in hydrogel matrices for various biomedical applications [36,37].

### 2.3. Clay Minerals Based Nanocomposite Hydrogels

The well-known limitations of conventional hydrogel systems include low mechanical strength, elasticity, and their non-interactive nature with organic fluids. Furthermore, they might be very sticky and their 3D structure can be destroyed under high pressure. However, the dispersion of organic mineral clays incorporates the best properties of both entities and widens their implicational area in biomedical sciences [38], such as in ideal wound/burn dressings [39]. The present section describes a few interesting features of clay minerals and their use in nanocomposite hydrogel systems.

The understanding of the role of clay minerals within a hydrogel system could be best understood by focusing on the structure of the clay mineral. Clay minerals having a layered structure for, e.g., montmorillonite, which is an alumino-silicate, have relatively high cation exchange capacity. For example, the replacement of natural exchange cations (sodium ion to alkylammonium) converts the clay surface characteristics from hydrophilic to hydrophobic, which creates a strong interaction of the clay with organic compounds dissolved in water/bodily fluids. These minerals get exfoliated, or intercalated, or both, depending on the loading of the minerals (low, moderate, or high) within the hydrogel. As studied, a linear stress-strain correlation was seen with increased concentration of clay minerals, which was similar to robust/elastic rubbers or chemical gels [40,41]. Their presence affected the swelling behavior and the selective adsorption resulted due to variation in structural integrity, physical, mechanical properties, viscosity, along with their ability to act as a physical barrier to microbe penetration inhibiting the bacterial/fungal growth. These hydrogel-clay mineral hybrid composites act as an extra cellular matrix, aiding in the dynamic regenerative process of wound healing. They involve a balanced, systematic coordination in inflammatory, vascular, connective tissue, and epithelial cells by protecting the wound from secondary infections, maintaining a moist environment, and simultaneously absorbing the wound fluids and controlling the drug release rate. All these conditions stimulate cellular growth and reduce wound necrosis [42]. Exfoliated or intercalated clay minerals can further form chemical bonds with other chemical crosslinkers to enhance mechanical stability, cell adhesion, and cellular growth. Particularly, cells such as mesenchymal stem cells, which are anchorage-dependent cells, are difficult to adhere to the substrate-like polyethylene glycol and lose cellular growth, spreading, and cell compatibility [43]. Thus, clay minerals preserve their great future as a nanofiller in hydrogels [44].

### 2.4. Fumed Silica Based Nanocomposite Hydrogels

Another well-known nanofiller used for various biomedical applications is fumed silica [45]. The various forms of silica have been explored for diverse applications. Pyrogenic silica has low bulk density, high surface area, as well as a three-dimensional structure. The nanoporous amorphous silica can increase their stability by being rearranged into long chains, branches, secondary, or tertiary structures with tunable properties. Both hydrophobic and hydrophilic properties can be induced during the synthesis of this material. They have been used as additives or as reinforcing agents in polymeric scaffolds, hydrogels, and other hybrid materials to improve physico-mechanical, physico-chemical, catalytic properties, and surface hydrophobicity [46,47,48,49]. The inclusion of silica has been exclusively helpful in inducing higher biodegradability, drug/dye adsorption, stimuli responsiveness, improved cell adhesion and proliferation, as well as selective detection of biomarkers [46,50,51,52]. Moreover, the novel properties developed in porous silica nanoparticles can be tuned in terms of morphology and surface functionalization, leading to their application in ophthalmic prostheses, vascular prostheses, drug delivery systems, and soft-tissue scaffolds, etc. [53]. Hydrogel hybrids composed of fumed silica and other nanostructures have proven to improve the stability (thermal, chemical, and mechanical) of the materials, for example, novel nanocomposite hydrogels were prepared using poly(hydroxyethylmethacrylate) (pHEMA) as organic moiety and fumed silica nanoparticles (3–40% *w*/*w* to the organic monomer) as inorganic fillers. These hybrids can act as scaffolds for bone engineering, as the primary cultures of human osteoblasts (OB) showed improved adhesion, cyto-compatibility and proliferation with an increase in the nanomeric filler content [52]. Furthermore, silica, owing to its three-dimensional structure, also acted as a crosslinker for natural as well as synthetic polymers, such as alginate, gelatin, chitosan, cellulose, polyacrylamide, poly(ethylene glycol), and poly(vinyl alcohol), etc. [54,55,56,57], thus holding great potential in the long run.

## 3. Biomedical Applications of Nanocomposite Hydrogels

### 3.1. Drug Delivery

Nanocomposite hydrogels have shown great potential in drug delivery. Concerns have been raised over the biocompatibility of developed nanocomposite hydrogels when inorganic nanofillers are used inside the hydrogel matrix. Nanofillers originated in 1980s. Toyota made the first development in nanoclay that has been used as a nanofiller [54]. Nanofillers, particularly based on clays and carbon nanotubes, gained wider consideration as they hold great potential for various biomedical and theranostics applications. They have prodigious prospective in terms of their effectiveness in reinforcing filler and significance with dissension in polymer matrices. These nanofillers are stronger, lighter, cost-effective, more versatile, and reliable [55]. They have remarkable potential when compared to polymer and carbon nanotubes for drug delivery systems [56]. A nanoclay particle reduces the porosity of a polymer, thereby improving the utilization of polymers. The exploration of clay-drug interaction and release mechanisms has had a critical impact on the development of formulations of clay-based drug delivery systems. Clay minerals are widely used to improve drug dissolution rate. Improving the dissolution of drugs remains one of the more crucial challenges for its biomedical applications. Possible mechanisms of interaction between drugs and clay are important to analyze, as they could provide insights for how to modify drug release. Recent strategies have shown improved drug stability and simultaneously modified the drug delivery outline by the usage of clay minerals [57,58]. On the basis of their high retention capacities, colloidal, and swelling properties, clays have shown their applicability in drug delivery [59,60]. There are natural clays, semi-synthetic, or synthetic derivatives to carry out precise functions in new drug delivery systems. Monkhouse and Lach et al. (1972) have demonstrated the use of silica and silicic acid as the adsorbent agents. Smectites were found to efficiently improve the in vitro dissolution rate of non-ionic and acidic insoluble drugs. Pharmaceutical grade clay minerals such as smectite, kaolinite, and fibrous clay minerals have been utilized for prolonged and slow drug release [61]. In another study, Koeleman et al. (1990) reported that phenytoin-montmorillonite adsorbates are capable if improving the bioavailability of a drug when compared to phenytoin sodium capsules in humans. Drug release from the clay surface is stimulated by a weaker bond, which concurrently results in drug-enhanced wettability, due to the hydrophilic properties of the clay [62]. Ito et al. (2001) showed in vivo that blood level of indomethacine from transdermal patches based on drug-montmorillonite composites was greater compared to control compounds containing crystalline indomethacine [63]. Boraie et al. (1986) reported that tablets prepared by direct compression of hydrochlorotiazide-veegum adsorbates showed complete dissolution (100%) of a drug in 10 min, however commercial tablets released 50% of the dose [64]. Koeleman et al. (1990) showed the influence of montmorillonite on the dissolution and bioavailability of phenytoin. The authors investigated the dissolution of phenytoin from various phentyoin-montmorillonite combinations, which were obtained by precipitating phenytoin from two different solvents and by mixing phenytoin and montmorillonite into compressed tablets. The dissolution rates were analyzed for phenytoin alone with the dissolution rates of the combinations. Interestingly, the montmorillonite were found to enhance the dissolution rate and bioavailability of phenytoin from all the combinations [62]. Their data validated the idea that combining phenytoin and montmorillonite upgrades the bioavailability of phenytoin. Nanoclays hold excessive medicinal applications for controlled release and delivery of various drugs. Alternative strategies are required to reduce the drug administration dosage and thereby the associated toxic side effects of drugs. Packaging biological cargoes in mesoporous materials provides efficient and effective opportunities for drug delivery. Passive approaches and active surface decoration techniques have been used for the generation of novel mesoporous silica nanoparticles (MSN) based drug delivery systems for targeted release [65]. Packaging biological cargoes in mesoporous materials and their potential scopes for drug delivery have been extensively review by Siefker et al. 2014 [66]. Mesoporous silica Santa Barbara Amorphous-15 (SBA-15) has remarkable and flexible pore diameter that offers a vast functionalized surface for efficient biomaterial utilization in biomedical applications. SBA-15 has potential as a carrier of biological therapeutics. There are studies that show the design and development of nanostructured mesoporous materials using SBA-15. These studies highlight its prospects for the delivery of therapeutic agents [67,68,69]. Several studies have also shown the utilization of carbon nanotubes (CNTs) for treatment of a various diseases. CNT-based anti-cancer drugs are one of such examples of CNTs’ biomedical application by selective targeting of specific tumor receptors and moreover, by the controlled release of drugs [70,71,72]. The ability of CNTs to specifically deliver drugs to tumor site with lesser toxicity and without any side effects makes them an ideal candidate with potential anticancer application properties. Cheng et al. (2011) developed a CNT-based anticancer drug that could solve the problem of multidrug resistant cancer cells and specifically target sensitive cancer cells [73].

### 3.2. Imaging and Gene Silencing

Nanocomposite hydrogels have essential properties that make them suitable for imaging applications. Especially when considering the limitation associated with conventional hydrogel systems, such as poor mechanical strength and rheological properties, nanocomposite hydrogels serve as the best nanocarriers in terms of mechanical strength, homogeneity of drug loading as well as targeting ability In this section, we highlights carbon nanotube-based nanocomposite hydrogel system nanofiller for imaging and gene silencing.

One interesting study has shown the development of nanocomposite hydrogels for unique ultrasound and imaging applications. The study involved the integration of preFITC-labeled functionalized nanolipobubbles within a crosslinked hydrogel network. Bioimaging techniques showed promising features of the developed hydrogels. Co-localization of the FITC-labeled nanolipobubbles after being embedded within the crosslinked network was observed. Confocal microscopy was used to confirm the distribution of the functionalized nanolipobubbles from the hydrogel into PC12 neuronal cells [74].

The unique physicochemical properties of CNTs make them a potential candidates for several applications in biomedical fields such as in imaging and gene silencing [75]. CNT properties, such as the basics of synthesis and purification of CNTs, have been extensively reviewed [76,77]. Sinha and Yeow (2005) discussed the challenges associated with CNTs, which need to be addressed for their efficient biomedical applications [76]. Carbon nanotubes (CNTs) possess exclusive physical, mechanical, and electronic properties. The great benefit of CNTs is that they can be loaded with a variety of biomolecules like siRNAs, genes, and DNA. This feature can make them an effective tool in gene silencing and gene delivery. CNTs can load several ranges of biomolecules with therapeutic relevance such as genes, siRNA, DNA, and aptamers [78,79]. This characteristic makes CNTs effective vehicles for gene delivery and also for gene silencing.

### 3.3. Orthopedic Applications

**Figure 3:**
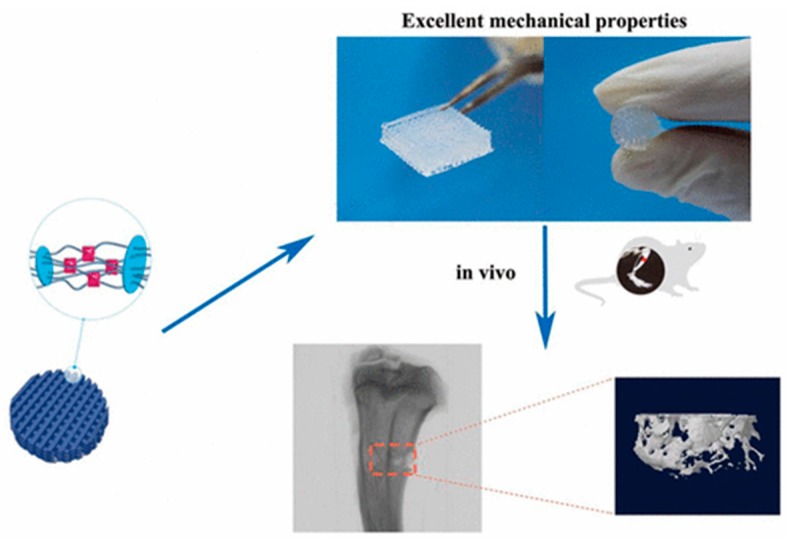
Supramolecular polymer/clay nanocomposite hydrogel scaffold for bone regeneration. Reprinted with permission from Reference [80]. Copyright 2017 American Chemical Society.

Hydrogels act as potential biomaterials and have attracted attention as scaffolds, especially in case of bone defects and other orthopedics applications. Emphasis has been placed on developing high-strength hydrogels for bone treatment. In this regard, nanocomposite hydrogels have emerged with great relevance, due to their resemblance to the extracellular matrix. One recent study showed the development of a hybrid bioink of hydrogen-bonding monomer (*N*-acryloyl glycinamide) (NAGA) and nanoclay. This hydrogel showed high mechanical properties (Figure 3) and swelling stability [80]. These gels showed the osteogenic differentiation of primary rat osteoblast (ROB) cells. This study showed that these gels were applicable in easing of the regeneration of the new bone in tibia defects in rats.

Various metal ions can be employed for better bone formation and growth. Magnesium Mg^2+^, has gained recent attention as a nanofiller in hydrogels which shows great cell adhesion and differentiation for bone development [81].

**Figure 4:**
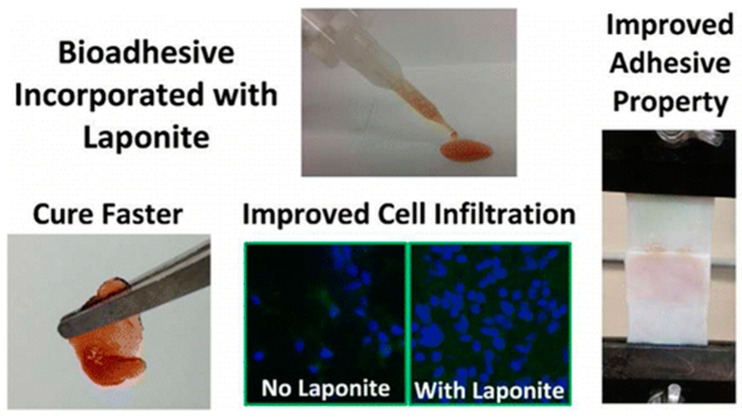
Injectable dopamine-modified poly(ethylene glycol) nanocomposite hydrogel with enhanced adhesive properties and bioactivity. Reprinted from Reference [82]. Copyright 2014 American Chemical Society.

Injectable forms of hydrogels are also being developed by the addition of nanofiller to form injectable nanocomposite hydrogels. One interesting study showed that the addition of laponite to the dopamine-modified four armed poly(ethylene glycol) resulted in the composite hydrogels having potentially high adhesive and mechanical properties. These hydrogels showed that subcutaneous implantation in rat exhibited minimum inflammatory response and enhanced cellular infiltration and fast curing (Figure 4) [82].

Nano-hydroxyapatite has also been explored in the development of nanocomposite hydrogels for bone regeneration, due to its superior biological properties and strong mechanical features. Polyacrylamide hydrogels, when combined with nano-hydroxyapatite, showed high fracture tensile stress, extensibility, and compressive strength [83].

### 3.4. Biosensing Applications

Biosensors detect and convert biological reactions to a signal, which is measurable by a variety of techniques including conductometric, potentiometric, amperometric, impedimetric, surface charge, piezoelectric, magnetoelastic, surface acoustic wave, fiber optic, acoustic wave, fiber optic, absorbance, and luminescence, etc. Hydrogel acts as a base material to form a biosensor connected to a bioreceptor that binds to a specific target. There are different bioreceptors including (1) antigen/antibodies, (2) enzymes, (3) cells and cellular structures (4), nucleic acids and DNA, and (5) biomimetic materials [84]. In the present section, we will brief few nanocomposite hydrogel system used for biosensing applications.

Adding organic components, e.g., polyaniline, as an electronic conductor to hydrogel results in a conducting polymer hydrogel (CPH). CPH as a biosensor showed high permeability to biomolecules, biocompatibility, and electron transfer properties [85]. Polypyrrole and poly(ethylenedioxy thiophene) have also been reported to be used as electronic conductors to fabricate CPH [84]. In a report by Vedrosa et al., gold nanoparticle-containing hydrogel was developed to fabricate electrochemical enzyme-based biosensors in which the prepolymer solution was photolithographically micropatterned in alignment with an array of gold electrodes fabricated on glass. The presence of gold nanoparticles enhanced the conductivity of PEG hydrogel by a factor of 5, which was confirmed by impedance measurements [86]. Q. Rong et al. also reported the fabrication of network nanocomposites based on polypyrrole hydrogel (PPy hydrogel) and gold nanoparticles. The electrodeposited gold nanoparticles on the PPy hydrogel increased both the specific surface area, to capture more antibodies, and electron transfer. This biosensor was employed for the fabrication of a sensitive label-free amperometric immunosensor [87].

Carbon nanotubes and graphene have also been used to enhance mechanical and electrical properties of conventional hydrogels for biosensor applications [88].

There are several advantages and applications of using biosensors based on CNTs. CNT biosensors have high sensitivity because of their hollow tubular structure and higher surface to volume ratio [89,90]. Additionally, CNTs can be used to immobilize enzymes. They have efficient and fast response time, due to their fast electron-transfer kinetics and also have lesser surface fouling effects, longer life span, and high stability. CNT-based nanocomposite hydrogel systems can be employed for biomedical imaging in order to examine and analyze cells, tissue, and organs with high resolution. In vivo experiments done by Vittorio et al. have demonstrated that CNTs can be specifically directed by an external magnetic source toward a chosen organ [91].

**Figure 5:**
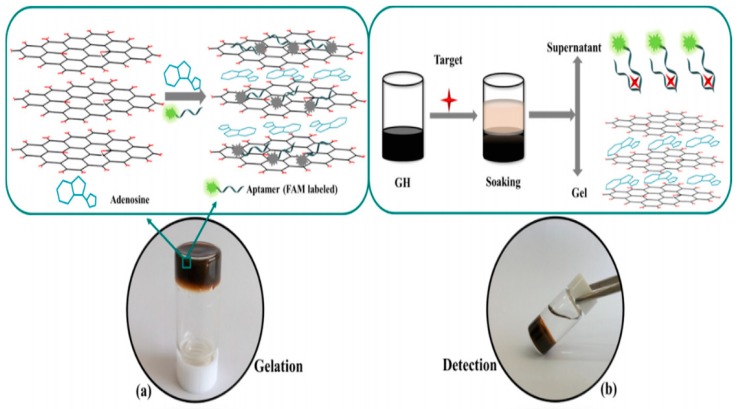
Fabrication of 3D macroscopic hydrogel with graphene oxide nanosheets (**a**) and the mechanism of selective detection of antibiotics (**b**). Reprinted with permission from reference [92]. Copyright 2016 Elsevier.

Graphene oxide hydrogel (GOH) has been developed and utilized as a fluorescent biosensor in antibiotic detection [92]. The GOH biosensors worked based on immersion and fluorescence determination processes and showed high mechanical strength and thermal stability. Graphene oxide hydrogels were prepared by mixing a solution of graphene oxide nanosheets with adenosine. GOH exhibited strong hydrogen bonding and electrostatic interactions between the adenosine and the graphene oxide. Aptamers, a functional single-stranded DNA (ssDNA), were added as a recognition element. The strong π–π stacking interactions between the hexagonal cells of graphene and the ring structure of nucleobases in ssDNA caused the ssDNA chains to lay on the surfaces of graphene oxide nanosheets and also was a driving force in assembling graphene oxide nanosheets into hydrogels. Figure 5 shows that adenosine and aptamer act as cross-linkers between the graphene oxide nanosheets, where aptamer is of a functional ssDNA or RNA, which can recognize and bind to their cognate targets.

Nanocomposite hydrogels composed of metal-oxide nanoparticles including iron oxides, titanium oxide (TiO_2_), alumina (Al_2_O_3_), and zirconia (Zr_3_O_2_) have also been explored for biosensing applications [6].

## 4. Conclusions

Nanocomposite hydrogels are the most innovative biomaterials emerging for various biomedical applications. These mechanically strong and superior hydrogels have diverse applications in the fields of optics, sensors, actuators, tissue engineering, and many others. These hydrogels have exhibited numerous benefits over conventional hydrogels. They have high physical strength, high conductivity, stiffness, and are very stable. The combination of the properties of hydrogel matrices with nanofillers makes them unique materials with diverse applications. The synergism that comes from the addition of inorganic fillers with organic polymers would lead to future materials with novel and improved properties. The association of organic and inorganic matrices produces beneficial features. Interestingly, the organic polymeric matrix controls the morphology and the swelling properties of the three-dimensional network, whereas the inorganic particles enhance the mechanical strength, thermal stability, optical properties, magnetic strength, bioactivity, and conductivity. There is an intense need to deploy green methods to tailor nanocomposite hydrogels so that they have lower toxicity and other side effects. The advanced technology demands to improvement of the properties of the existing nanocomposite systems for medicinal and biosensing applications. This technology is emerging with development of bionanocomposites and producing outstanding results in medicine.
